# Light-powered, artificial molecular pumps: a minimalistic approach

**DOI:** 10.3762/bjnano.6.214

**Published:** 2015-11-02

**Authors:** Giulio Ragazzon, Massimo Baroncini, Serena Silvi, Margherita Venturi, Alberto Credi

**Affiliations:** 1 Photochemical Nanosciences Laboratory, Dipartimento di Chimica “G. Ciamician”, Università di Bologna, via Selmi 2, 40126 Bologna, Italy; 2 Interuniversity Center for the Chemical Conversion of Solar Energy (SolarChem), Bologna Unit, via Selmi 2, 40126 Bologna, Italy,; 3 Istituto per la Sintesi Organica e la Fotoreattività, Consiglio Nazionale delle Ricerche, via Gobetti 101, 40129 Bologna, Italy

**Keywords:** azobenzene, molecular machine, photochemistry, rotaxane, supramolecular chemistry

## Abstract

The realization of artificial molecular motors capable of converting energy into mechanical work is a fascinating challenge of nanotechnology and requires reactive systems that can operate away from chemical equilibrium. This article describes the design and construction of a simple, supramolecular ensemble in which light irradiation causes the directional transit of a macrocycle along a nonsymmetric molecular axle, thus forming the basis for the development of artificial molecular pumps.

## Review

### Introduction

Since ancient times, man has tried to construct devices that facilitate life. With the advent of nanoscience and nanotechnology, which aim at controlling matter on the nanometer scale, chemists have started to develop devices of molecular size capable of performing simple functions [1]. In this regard, an important source of inspiration and knowledge is represented by the natural world [2]. Indeed, owing to the progress in molecular biology, we know that living beings are endowed with biomolecules that can replicate genetic material, transport substances inside cells or across membranes, and can be switched on and off in response to external stimuli [3]. Other classes of proteins, appropriately organized in arrays such as muscle fibers, take care of the macroscopic movements of organisms. Albeit still far from the complexity and functionality of biological systems, chemists have begun to undertake the design and construction of simple molecular devices, and to understand their operational mechanisms [4–7], particularly with regard to the use of light as the source of energy [8–9]. As a matter of fact, molecular machines cannot be viewed merely as miniaturized versions of the corresponding macroscopic devices because several intrinsic properties of nanoscale systems are radically different from those of the macroscopic objects that we deal with in everyday life [10].

The research field of artificial molecular machines, which began in the early 1990s [11], has rapidly grown and has currently reached a significant degree of maturity, as witnessed by the publication of monographies [4], books [12], many review articles [5–8,10], and hundreds of scientific papers. In the past decade, the device-driven ingenuity of chemists and the use of increasingly sophisticated methodologies for molecular synthesis and characterization allowed the realization of a wide range of nanoscale devices, including mechanical switches [13–14], tweezers [15], valves [16], gyroscopes [17], elevators [18], and linear [19] and rotary [20] motors.

**Figure 1:**
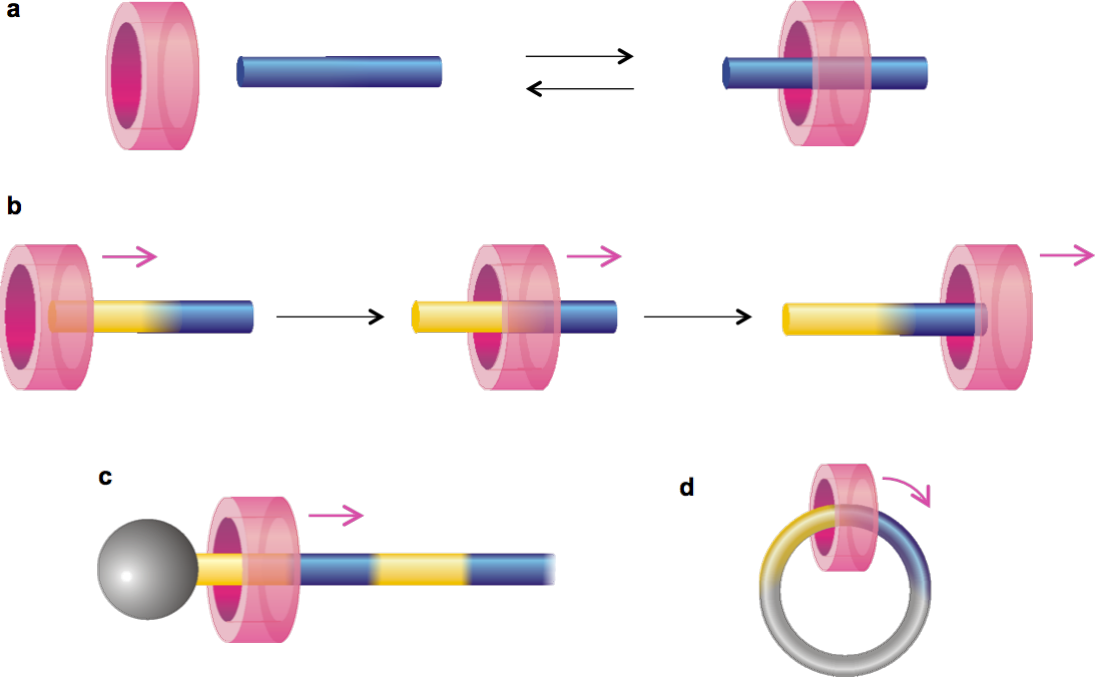
Schematic representation of the threading/dethreading of a pseudorotaxane (a) and of the relative unidirectional transit of a macrocycle along a nonsymmetric molecular axle (b). By incorporating the system shown in (b) in a rotaxane or a catenane, linear (c) or rotary (d) motors may be obtained, respectively.

Multicomponent molecular systems such as rotaxanes and related species are frequently used to realize molecular devices and machines [4–8]. In their simplest form, rotaxanes are composed of a thread-like molecule (the axle) surrounded by a macrocyclic molecule (the ring) and terminated at both ends with bulky groups (the stoppers) that prevent the components from disassembling. If the thread-like component does not have stoppers, the resulting ring–axle complex, termed pseudorotaxane, is in equilibrium with the separated molecular components in solution. The formation of the pseudorotaxane is enabled by the presence of noncovalent interactions between the ring and the axle, which can be modulated by an external stimulus, such that the threading and dethreading of the molecular components can be controlled (Figure 1a). Studies on switchable pseudorotaxanes form the basis of the field of artificial molecular machines and are a necessary premise for the realization of more sophisticated systems.

Today, the stimulus-controlled threading and dethreading of pseudorotaxanes are well known processes that have been realized with a wide variety of chemical structures [4–8]. They involve the ability to adjust the thermodynamic stability of the complex with respect to the separated molecular components. This, in turn, is related to the modulation of the noncovalent intercomponent interactions. Far less investigated are strategies that cause the threading/dethreading to occur along a specific direction, that is, to obtain the unidirectional transit of the ring along a nonsymmetric molecular axle, as shown in Figure 1b [21]. Such strategies require both thermodynamic and kinetic control over the threading and dethreading processes and are the basis of the construction of linear (Figure 1c) and rotary (Figure 1d) molecular motors.

Our group recently developed a simple, self-assembling system in which a macrocycle is displaced unidirectionally along a nonsymmetric molecular axle in a repetitive fashion using light as the sole energy source [22]. The system, which operates autonomously (that is, under continuous experimental conditions and without external intervention, as long as the light source is on) and does not generate byproducts, is the first example of an artificial molecular pump operated by light. A pseudorotaxane-based supramolecular pump operated by redox reactions in solution was recently reported [23–24]; this system, however, is not autonomous and consumes chemical fuels. The scope of this article is to introduce the concept of the autonomous molecular motor, to discuss the design principles for the continuous conversion of light energy into directed motion, and to illustrate the experimental steps that led to the realization of a photochemical molecular pump.

### From molecular switches to autonomous molecular motors

**Figure 2:**
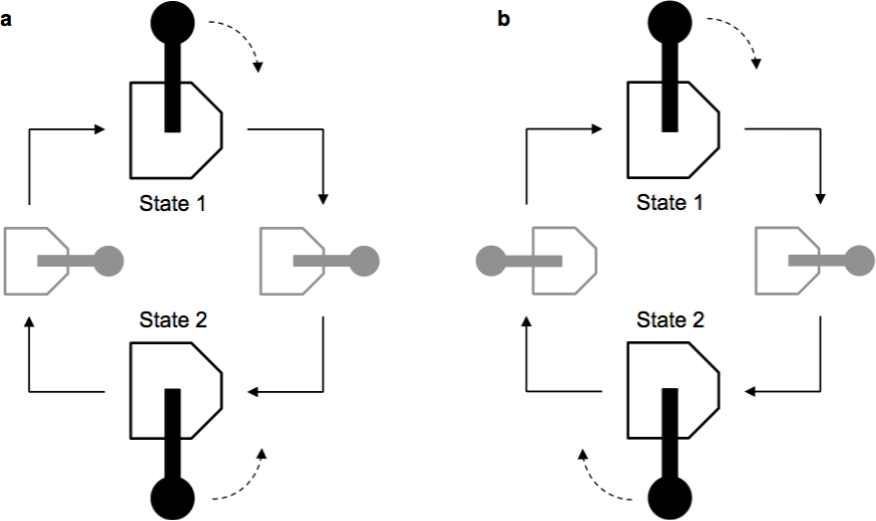
In a switch (a) the interconversion between states 1 and 2 takes place through the same transformation performed in opposite directions. In a motor (b) the conversion from 1 to 2 and from 2 to 1 occurs by following different routes.

The vast majority of molecular devices studied so far belong to the class of molecular switches. A molecular or supramolecular system behaves as a switch if it can exist in at least two different forms, each characterized by given physical and chemical properties, which can be reversibly converted into one another in consequence of the application of an external stimulus. In a switch, the transformation that leads from state 1 to form 2 follows a path that is the exact reverse of the transformation from 2 to 1 (Figure 2a). This feature has an important physical implication, that is, the cyclic interconversion between the states of a molecular switch cannot be exploited to perform work on the system such that it will be pushed progressively away from equilibrium.

To understand the meaning of this statement we can use an example from everyday life. Let us imagine a person using a pulley to lift a weight by repeatedly exchanging the relative position of the hands with respect to the rope between state 1 (left hand ahead, right hand behind) and state 2 (right ahead, left behind). The operation of the switch can be compared to the situation in which the person has one hand bound to the rope: irrespective of the exerted force, the weight will not be lifted because the exchange in the position of the hands will put down the load by the same amount that has just been pulled up. To reach the goal the operator needs to pull the rope with one hand and move the other hand forward, alternating hands such that the work previously done is not cancelled out. This means that the conversion between states 1 and 2, each corresponding to a different hand configuration relative to the rope, must take place by following nonequivalent routes. Similarly, in a molecular motor, the transformation between the two mechanical states occurs through different configurations (Figure 2b) so that the work done in the first part of the cycle is not canceled in the second part.

Molecular motors, like macroscopic ones, need an energy source to operate. In reference to Figure 2, the transformation from state 1 to 2 requires an energy input; the same applies to the transformation from state 2 to 1, whose energy input should be alternated with the previous one so that the system can work cyclically. Such transformations can be caused by pH changes obtained by addition of acids and bases, or by redox reactions performed by adding oxidants and reductants. In these cases, the cyclical operation involves the repeated addition of reactants with the concomitant generation of waste products. At some point, this may compromise the integrity of the system unless they are removed, as occurs in natural molecular motors as well as in conventional combustion engines.

Light is an energy source of great interest for use with molecules that has been widely exploited to operate molecular switches for a long time. For example, in photochromic compounds [25], the transformations between the two states can be caused by light of different wavelengths, or one transformation is caused by light while the other takes place in the absence of light. Light has a number of advantages compared with other types of stimuli in the context of molecular devices [26]. Since light is a “reactant” that contains energy but no mass, it is capable of transforming substances in a clean manner, that is, without generating byproducts. Moreover, in some instances, the same photons can induce both the conversion from state 1 to 2 and its reverse. A molecular machine exhibiting this property is of high interest because it can operate in an autonomous fashion, that is, under stationary experimental conditions and with no external intervention, as long as the light energy is supplied.

### Modulation of the potential energy profiles

This work was developed with the goal of obtaining the relative, unidirectional transit of a macrocycle along a molecular thread using a minimalistic approach; in other words, to identify the simplest molecular structures capable of realizing a mechanism based on minimal functional requirements. The developed strategy relies on the use of a crown ether as the macrocyclic ring and a nonsymmetric molecular axle with three units: a terminal, photosensitive azobenzene group (A), an ammonium ion that performs as the recognition site (R) for the macrocycle, and a nonphotoactive unit (S). Azobenzene, which is stable in its *trans* configuration, can be temporarily converted in a photochemical manner into the metastable *cis* isomer, endowed with significantly different steric and electronic properties [27]. In particular, the threading of the *trans*-A unit through the crown ether ring is much faster than that of the *cis*-A unit [28]. The S moiety is chosen so that its hindrance for the passage of the macrocycle falls in between that of the *trans*-A (linear) and *cis*-A (bent) units.

**Figure 3:**
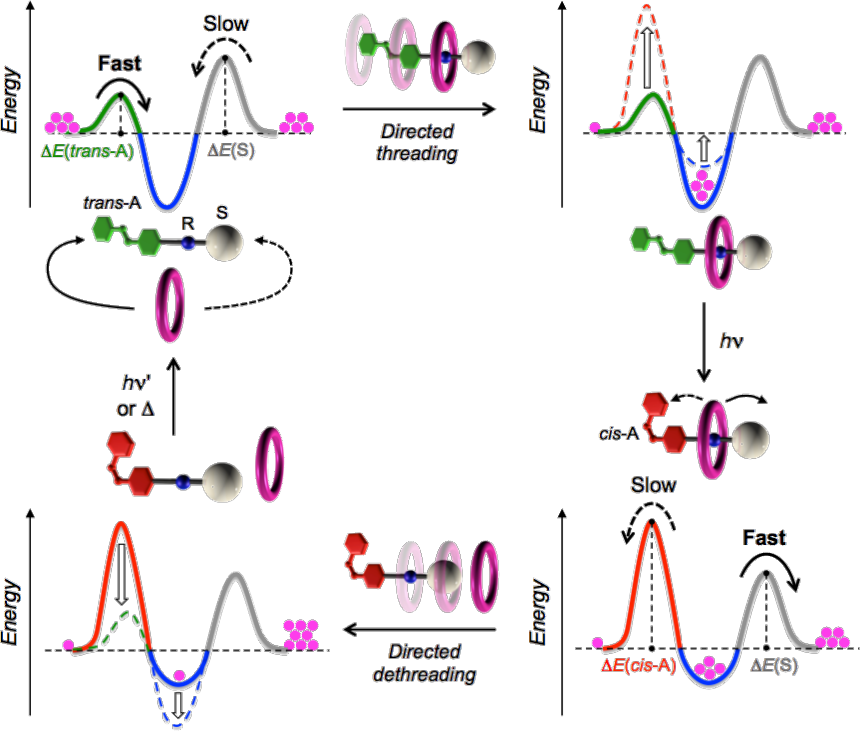
A minimalistic strategy for the photoinduced transit of a macrocycle along a nonsymmetric molecular axle. A simplified representation of the potential energy profile as a function of the ring–axle distance is shown for each state of the cycle. The periodic modulation of the potential energy maxima and minima, induced by light, enables the directionally controlled threading and dethreading of the molecular components.

The investigated strategy is shown in Figure 3. Because of the hydrogen bonds established between the molecular ring and the R unit located on the axle, a pseudorotaxane is formed in solution [29–30]. This process involves the passage of the ring on the *trans*-A unit because it is less hindered in comparison with the S unit. At this point, light irradiation in the ultraviolet or blue regions converts azobenzene into the *cis* form. This transformation causes, on the one hand, the destabilization of the pseudorotaxane by weakening the ring–axle interactions (increase of the energy minimum) and, on the other hand, the increase of the barrier that the ring has to overcome to pass along the *cis*-A unit, which has now become larger than that associated with the passage on the S unit (Figure 3). The thermal or photochemical transformation of *cis*-azobenzene into the stable *trans* form, which is clean and efficient, completes the cycle, regenerating the starting pseudorotaxane.

**Figure 4:**
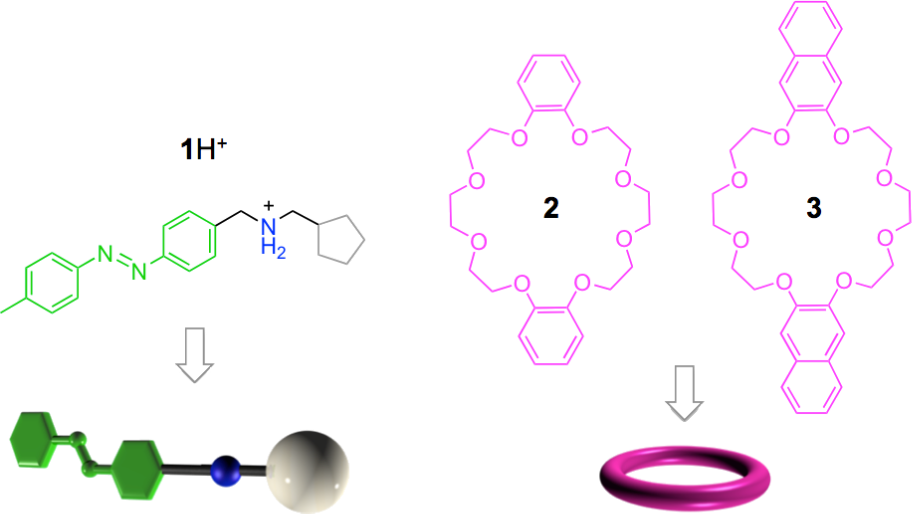
Structure formula and schematic representation of the examined molecular components.

A literature search [31] and molecular modelling experiments suggested that a good candidate for the critical role of the photoinactive pseudo-stopper S is the cyclopentyl moiety. The nonsymmetric molecular axle **1**H^+^ (Figure 4) was thus synthesized, and the photoinduced transit of the dibenzo-24-crown-8 ether **2** was investigated in acetonitrile at room temperature by steady state and time-resolved ^1^H NMR spectroscopy and UV–vis absorption experiments [32]. The kinetic measurements demonstrated that the photoisomerization of the A unit of **1**H^+^ determines the threading/dethreading side (i.e., the height of the energy maximum) but, unfortunately, it does not affect the affinity of the axle for the macrocycle (i.e., the energy minimum). In other words, for these compounds under the employed conditions, the dissociation of the ring from the macrocycle cannot be obtained by light irradiation.

To overcome this problem, a second stimulus was used, namely, the addition of K^+^ ions capable of competing with **1**H^+^ for the cavity of macrocycle **2**. Indeed, the experimental data showed that the addition of an excess of KPF_6_ to a solution of the 
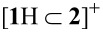
pseudorotaxane caused the extrusion of the ring from the S extremity of the axle, thus completing the directionally controlled threading/dethreading cycle [29]. Interestingly, the ring–axle interactions in 
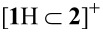
can also be switched off by deprotonating the ammonium site R with a base. This process, however, causes a significant increase of the energy of the system, such that the consequent dethreading is very fast, even if the ring has to pass along the azobenzene in the *cis* configuration [22].

### Autonomous operation under the effect of light

Building on these encouraging results, we improved the system so that the directional transit could be caused solely by light irradiation [18]. To this aim, the benzene units of macrocycle **2** were replaced with naphthalene ones (ring **3**, Figure 4). Such naphthalene units are strongly fluorescent and thus enable the use of luminescence spectroscopy (a more sensitive technique than the NMR methods used in earlier experiments) to study the threading and dethreading processes. We also anticipated that the presence of larger aromatic units in the molecular ring could amplify the difference in affinity of **3** for the two isomeric forms of the axle **1**H^+^ by exploiting the different ability of the *trans*- and *cis*-azobenzene units to interact with the naphthalene moieties by π-stacking. Finally, in order to enhance the ring–axle interactions, dichloromethane was used as the solvent in the place of acetonitrile. Indeed, spectrophotometric and fluorimetric titrations showed that the ring–axle association constants increased by almost three orders of magnitude in comparison to the values found in acetonitrile. Moreover, the association constant (*K*) of macrocycle **3** with axle *trans***-1**H^+^ (*K_trans_* = 6.3 × 10^5^ M^−1^) resulted to be about four times larger than that with the *cis*-axle (*K_cis_* = 1.7 × 10^5^ M^−1^). At the same time, the correct order of the rate constants (*k*) and, thus, the energy barriers (Δ*E*) associated with the passage of the macrocycle on the *trans*-A (*k_trans_*_-A_ = 54 M^−1^ s^−1^, Δ*E_trans_*_-A_ = 14.8 kcal mol^−1^), S (*k*_S_ = 0.81 M^−1^ s^−1^, Δ*E*_S_ = 16.7 kcal mol^−1^) and *cis*-A (*k_cis_*_-A_ = 3.9 × 10^−2^ M^−1^ s^−1^, Δ*E_cis_*_-A_ = 19.0 kcal mol^−1^) units was maintained, in agreement with the fact that the cavities of macrocycles **2** and **3** have an identical size [22].

**Figure 5:**
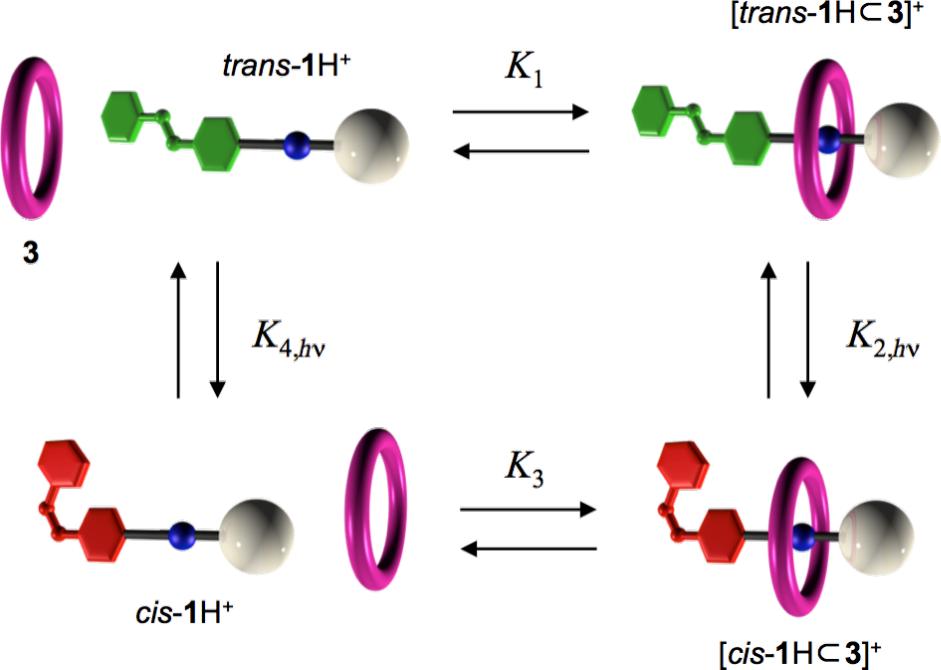
Self-assembly chemical reactions (horizontal processes) and photochemical isomerization reactions (vertical processes) that describe the operation of the molecular pump. The chemical or photochemical equilibrium constants refer to reactions read from left to right and from top to bottom. Figure adapted with permission from [18], copyright 2006 American Chemical Society.

Hence, in the new generation system, photoisomerization controls both the height of the kinetic barrier and the stability of the pseudorotaxane, as required for the strategy illustrated in Figure 3. The processes that describe the behavior of the device are represented in Figure 5, which highlights the correlation between the chemical threading/dethreading equilibria (horizontal processes) and the photoisomerization reactions (vertical processes). Our measurements demonstrated that under continuous light irradiation, the cycle shown in Figure 5 is travelled with higher probability in the clockwise direction than in the counterclockwise one. As is well known, the principle of microscopic reversibility states that in any closed path of chemical reactions, the cycling probability is the same in both directions, unless energy is introduced into the system, as it happens in the present case in the form of light [33]. The supply of energy, however, is not a sufficient condition to bring the system out of equilibrium.

In the next section we will provide an intuitive explanation on how this system can operate autonomously and how it can use (or, more precisely, dissipate) light energy to reach a nonequilibrium state. For this purpose, it is important to note that in a photoisomerization process (such as that of azobenzene), once a steady state is reached under light irradiation (photostationary state), the ratio between the amounts of the two isomers is constant. The composition of the system at the photostationary state can be expressed with the ratio of the concentrations of the two isomers (*K*_2,hν_ and *K*_4,hν_ in Figure 5). This represents a quantity mathematically equivalent to the equilibrium constant but with a different physical meaning, because it is not referred to a thermodynamic equilibrium state.

By analyzing the reaction cycle shown in Figure 5, one can notice that the transition from the state in which the ring and *trans*-axle are separated (top left) to that in which the ring and *cis*-axle are associated (bottom right) can be performed by following two alternative paths. The first one (clockwise half-cycle) corresponds to the formation of the *trans*-complex and its conversion to the *cis* form, whereas the second one (counterclockwise half-cycle) corresponds to the conversion of the free axle from *trans* to *cis* and subsequent formation of the complex. In a cycle of reactions at thermal equilibrium, the relative stability of the initial and final states is expressed by the product of the thermodynamic constants of the reactions that link the two states and must be independent on the followed path. This condition, with respect to the cycle presented in Figure 5, would result in the following expression:

[1]
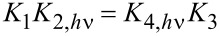


The experimental results indicate that under light irradiation at λ > 400 nm, the photostationary states of **1**H^+^ in the presence or in the absence of ring **3** have the same composition. In other words, *K*_2,_*_h_*_ν_ = *K*_4,_*_h_*_ν_. Since the titrations experiments show that *K*_1_ > *K*_3_, Equation 1 cannot be fulfilled and the *cis* complex is apparently more stable when it is generated through the clockwise half-cycle. Consequently, under steady light irradiation, the system reaches a stationary state (that is, a state in which the concentrations of all species are constant) corresponding to the cycle of Figure 5 preferrentially travelled clockwise.

The direct experimental observation of photostationary cycling is not straightforward because the physico-chemical properties of the ensemble are constant at steady state. As discussed above, however, a net flux around the cycle should be established by the supply of light energy even if the concentration of each species remains static, thus bringing the system out of equilibrium. Hence, the fact that the photostationary concentration for any species involved in the cycle is not consistent with its expected equilibrium value constitutes proof of autonomous, light-powered cycling. In this context, it is worth noting that the fluorescence intensity of **3** is completely quenched when it is complexed by either *trans***-** or *cis***-1**H^+^; therefore, the amount of uncomplexed macrocycle can be determined from the fluorescence intensity of the solution, measured under suitable spectral conditions [34]. Indeed, we found that upon light irradiation the concentration of the uncomplexed ring **3** shifts away from the range of values compatible with thermal equilibrium. When the light is switched off, the concentration of **3** slowly goes back to the same equilibrium value measured before illumination.

In summary, on the basis of the previous discussion (Figure 3 and Figure 5), the absorption of photons promotes the unidirectional, repetitive and autonomous transit of macrocycle **3** along axle **1**H^+^, thus realizing the first example of an artificial molecular pump activated by light. As already evidenced, the element that breaks the symmetry and enables the autonomous operation is the different stability of the ring–axle complexes in the two isomeric forms (*K*_1_ > *K*_3_). Furthermore, detailed measurements showed that for specific irradiation wavelengths, the photoisomerization efficiency is affected by complexation, namely, the photoreactivity of the axle depends on whether it is surrounded by the ring or not.

For example, the photostationary *cis*/*trans* ratio upon 365 nm irradiation is slightly larger for the complex than for the free axle, which means that *K*_2,_*_h_*_ν_ > *K*_4,_*_h_*_ν_. On the other hand, we observed that the photoisomerization quantum yield values are the same for the free and complexed axle. Thus, the larger *trans→cis* conversion in the complex must be due to the fact that its molar absorption coefficient at 365 nm is higher than that of the free axle. When the irradiation light is also absorbed by the ring component (e.g., at λ = 287 nm), the situation becomes even more interesting. Irradiation of the ring–axle mixture at 287 nm generates a photostationary state with a larger *cis*/*trans* ratio than for the axle alone (*K*_2,_*_h_*_ν_ > *K*_4,_*_h_*_ν_), as discussed above for excitation at 365 nm. Also, upon irradiation at 287 nm, the photoisomerization quantum yield of the axle is not affected by the presence of the ring. Thus, the reason why *K*_2,_*_h_*_ν_ is larger than *K*_4,_*_h_*_ν_ is because the excitation energy of **3** is effectively transferred to *trans***-1**H^+^ but not to *cis***-1**H^+^ in their respective complexes. This is due to a much better spectral overlap in the [*trans***-**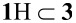]^+^ complex than in the [*cis***-**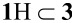]^+^ one.

These data enable an insightful discussion about the mechanisms used by this system to rectify Brownian fluctuations [10]. If irradiation is carried out with λ > 400 nm (*K*_2,_*_h_*_ν_ = *K*_4,_*_h_*_ν_), the mechanism is energy ratcheting, whereas upon irradiation at 365 or 287 nm, information ratcheting is also present. Different physical phenomena, however, account for the information ratcheting effect at these two wavelengths. The 365 nm light is not absorbed by the ring, but the presence of the latter enhances the absorption of the *trans*-axle, causing the preferential, photoinduced closure of the azobenzene gate in the pseudorotaxane with respect to the axle. The excitation of the ring with 287 nm light is followed by energy transfer to the *trans***-**axle, a process that can effectively take place only if the components are associated, and causes the *trans→cis* isomerization. Hence, upon irradiation at either 287 or 365 nm, the information about the relative position of the ring and axle components (i.e., whether they are associated or not) contributes to the gating.

From the analysis of the free energy change of the system, it can be estimated that the maximum amount of work that could be performed by the pump in a cycle under the employed conditions is 5.1·*k*_B_*T* (3.0 kcal mol^−1^ at 20 °C), which is about one fourth of the energy of ATP hydrolysis utilized by natural molecular motors. It can also be calculated that the system consumes on average 430 photons of 365 nm light (78 kcal mol^−1^) to complete a cycle, corresponding to an upper limit for the energy conversion efficiency of 9 × 10^−5^. Despite such a low conversion efficiency, this study clearly demonstrates that the synergy between photochemical reactions and self-assembly processes can lead to innovative methods for the conversion of sunlight into chemical energy [35].

## Conclusion

The most recent progress in the area of molecular machines has revealed the importance of designing and realizing molecular and supramolecular systems that can operate continuously away from equilibrium. The development of an artificial molecular pump powered by light following a minimalistic approach is an important step towards this goal and enabled the study and detailed understanding of some fundamental principles underlying the operation of molecular motors.

The structural simplicity and synthetic accessibility of the employed components are indeed important elements to favor the progress of this research and its possible applications. The present system, however, has a few important limitations. For example, because of the symmetry of the crown ether used as the macrocycle, it is impossible to establish which side of the ring cavity is pierced by the axle. Moreover, the device in its current form does not perform work but merely dissipates the light energy input into heat. The main challenge for this research is to modify the system so that the molecular movements can be used to generate concentration gradients or to transport chemical species along predetermined directions for significant distances in comparison with typical diffusive paths in solution.

## References

[1] Browne W R, Feringa B L (2006). Nat Nanotechnol.

[2] Jones R A L (2008). Soft machines: nanotechnology and life.

[3] Mann S (2008). Angew Chem, Int Ed.

[4] Balzani V, Credi A, Venturi M (2008). Molecular devices and machines: Concepts and perspectives for the nanoworld.

[5] Kay E R, Leigh D A, Zerbetto F (2007). Angew Chem, Int Ed.

[6] Abendroth J M, Bushuyev O S, Weiss P S, Barrett C J (2015). ACS Nano.

[7] Kay E R, Leigh D A (2015). Angew Chem, Int Ed.

[8] Silvi S, Venturi M, Credi A (2011). Chem Commun.

[9] Raiteri P, Bussi G, Cucinotta C S, Credi A, Stoddart J F, Parrinello M (2008). Angew Chem, Int Ed.

[10] Astumian R D (2007). Phys Chem Chem Phys.

[11] Anelli P L, Spencer N, Stoddart J F (1991). J Am Chem Soc.

[12] Credi A, Silvi S, Venturi M (2014). Molecular Machines and Motors: Recent Advances and Perspectives.

[13] Avellini T, Li H, Coskun A, Barin G, Trabolsi A, Basuray A N, Dey S K, Credi A, Silvi S, Stoddart J F (2012). Angew Chem, Int Ed.

[14] Bleve V, Schäfer C, Franchi P, Silvi S, Mezzina E, Credi A, Lucarini M (2015). ChemistryOpen.

[15] Leblond J, Petitjean A (2011). ChemPhysChem.

[16] Saha S, Leug K C-F, Nguyen T D, Stoddart J F, Zink J I (2007). Adv Funct Mater.

[17] Garcia-Garibay M A (2005). Proc Natl Acad Sci U S A.

[18] Badjic J D, Ronconi C M, Stoddart J F, Balzani V, Silvi S, Credi A (2006). J Am Chem Soc.

[19] von Delius M, Geertsema E M, Leigh D A (2010). Nat Chem.

[20] Conyard J, Addison K, Heisler I A, Cnossen A, Browne W R, Feringa B L, Meech S R (2012). Nat Chem.

[21] Arduini A, Bussolati R, Credi A, Monaco S, Secchi A, Silvi S, Venturi M (2012). Chem – Eur J.

[22] Ragazzon G, Baroncini M, Silvi S, Venturi M, Credi A (2015). Nat Nanotechnol.

[23] Cheng C, McGonigal P R, Schneebeli S T, Li H, Vermeulen N A, Ke C, Stoddart J F (2015). Nat Nanotechnol.

[24] Cheng C, McGonigal P R, Stoddart J F, Astumian R D (2015). ACS Nano.

[25] Irie M (2000). Photochromism: Memories and Switches. Chem Rev.

[26] Ceroni P, Credi A, Venturi M (2014). Chem Soc Rev.

[27] Bandara H M D, Burdette S C (2012). Chem Soc Rev.

[28] Baroncini M, Silvi S, Venturi M, Credi A (2010). Chem – Eur J.

[29] Ashton P R, Campbell P J, Glink P T, Philp D, Spencer N, Stoddart J F, Chrystal E J T, Menzer S, Williams D J, Tasker P A (1995). Angew Chem, Int Ed Engl.

[30] Rogez G, Ribera B F, Credi A, Ballardini R, Gandolfi M T, Balzani V, Liu Y, Northrop B H, Stoddart J F (2007). J Am Chem Soc.

[31] Ashton P R, Baxter I, Fyfe M C T, Raymo F M, Spencer N, Stoddart J F, White A J P, Williams D J (1998). J Am Chem Soc.

[32] Baroncini M, Silvi S, Venturi M, Credi A (2012). Angew Chem, Int Ed.

[33] Astumian R D (2012). Nat Nanotechnol.

[34] Credi A, Prodi L (2014). J Mol Struct.

[35] Balzani V, Credi A, Venturi M (2008). ChemSusChem.

